# Computation of Exchange Couplings by Means of an Exchange-Dedicated Perturbation Theory

**DOI:** 10.1021/acs.jctc.5c00733

**Published:** 2025-09-04

**Authors:** Michael Franz, Frank Neese, Sabine Richert

**Affiliations:** † 9174 Institute of Physical Chemistry, University of Freiburg, Albertstraß e 21, 79104 Freiburg, Germany; ‡ Department of Molecular Theory and Spectroscopy, 28314 Max-Planck-Institut für Kohlenforschung, Kaiser-Wilhelm-Platz 1, 45470 Mülheim an der Ruhr, Germany

## Abstract

The accurate computation of high-spin/low-spin gaps remains a challenging task in computational chemistry, with significant implications for both theoretical studies and experimental applications. In this work, we present an exchange-dedicated perturbation theory (EDPT2) that allows an efficient calculation of exchange couplings in magnetic systems. Our approach builds on a previously developed second-order perturbative scheme based on de Loth’s formalism but refines the treatment of singlet wave functions by explicitly incorporating ionic determinants in the zeroth-order description. The EDPT2 method is derived from a two-electron-two-center model and can be applied to multispin systems using minimal CAS-generated orbitals. A key advantage of EDPT2 lies in its computational efficiency, with a scaling of *N* ^4^, where *N* is the number of basis functions. Benchmark calculations on diverse test systems demonstrate that EDPT2 achieves high-spin/low-spin gaps with accuracy comparable to the commonly used FIC-NEVPT2 method. Beyond its efficiency, EDPT2 provides valuable information on the mechanisms that govern magnetic exchange. The method allows for a detailed decomposition of second-order contributions, facilitating the identification of dominant exchange pathways. This is exemplified on two bis­(nitronyl nitroxide) biradicals, where dynamic spin polarization emerges as the key exchange mechanism. Furthermore, using the example of a trisnitroxide triradical, we demonstrate how the insights from EDPT2 can be used to prepare selective multireference CI approaches. A combined DDCI1 approach with EDPT2-derived corrections is shown to successfully reproduce the experimental doublet-quartet gap.

## Graphical abstract

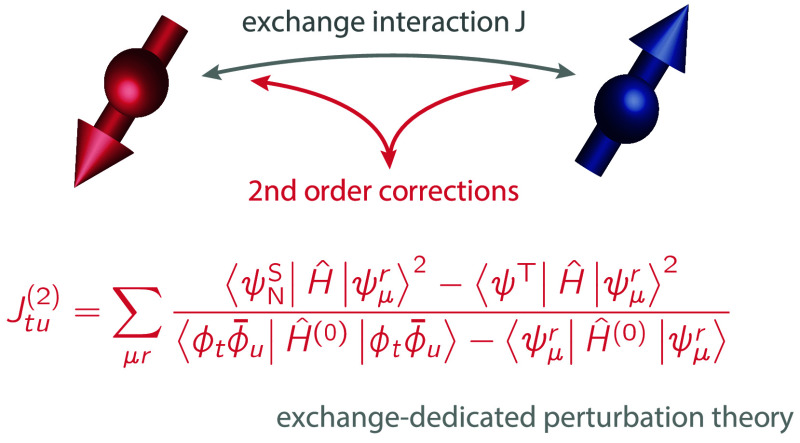

## 1 Introduction

The computation of high-spin/low-spin gaps is a challenging task within the field of computational chemistry and is of interest not only to theoreticians but also to experimental chemists. The energetic gap between high-spin and low-spin states can be described as a function of exchange interactions, *J*_*tu*_, for example, by using the isotropic Heisenberg–Dirac-van-Vleck model Hamiltonian *Ĥ* ^HDVV^ = Σ*_t_*_<*u*_ – *J*_*tu*_ *Ŝ*_*t*_ *Ŝ*_*u*_ assuming the orbitals *t* and *u* to be localized on the magnetic centers.^1–5^ One may fit experimental measurements, for example, by SQUID or EPR, onto such a model Hamiltonian in order to gain access to high-spin/low-spin gaps.^1^ However, the experimental determination of the exchange coupling is not always possible, as is often the case for compounds where the magnetic coupling arises from an excited state.^6^ Here, the computational determination of the magnetic coupling becomes particularly attractive. In addition to computing the magnitude of the magnetic coupling, it is also possible to gain insight into the mechanisms underlying the magnetic coupling.^1,5,7–12^

Over half a century, different approaches were developed to compute and rationalize the exchange interaction. One may mention qualitative valence-only models, such as the Kahn-Briat model^13,14^ and the Hay-Thibeault-Hoffmann model^15^ or the second-order perturbational approach by de Loth,^16^ which is based on a theorem stated by Malrieu.^17^

In a previous study, we developed a method based on the work of de Loth, where the individual exchange couplings *J*_*tu*_ are computed by means of second-order corrected singlet–triplet gaps using orbitals obtained from complete active space self-consistent field^18,19^ (CASSCF) calculations with minimal active spaces.^8^ To describe the singlet and triplet states, we considered a neutral subspace spanned by the Slater determinants |*ϕ_t_ϕ̅*_*u*_⟩ and |*ϕ_u_ϕ̅*_*t*_⟩. The resulting singlet and triplet states are then corrected up to the second order only by those excited determinants which can interact simultaneously with the reference determinants |*ϕ_t_ϕ̅*_*u*_⟩ and |*ϕ_u_ϕ̅*_*t*_⟩.^8,16^ Such a procedure can be carried out very efficient due to the limited excited determinant space and gives insights into the magnetic coupling by means of the individual contributions arising from specific groups of excited determinants.^16,20–23^ However, by neglecting the ionic determinants |*ϕ_u_ϕ̅*_*u*_⟩ and |*ϕ_t_ϕ̅*_*t*_⟩ in the zeroth-order description of the singlet wave function, one misses larger contributions to the exchange coupling for increasing amplitudes of |*ϕ_u_ϕ̅*_*u*_⟩ and |*ϕ_t_ϕ̅*_*t*_⟩ in the zeroth-order wave function of the singlet state.^1,5,16^ In this work, we refine our previous approach by including the ionic determinants |*ϕ_u_ϕ̅*_*u*_⟩ and |*ϕ_t_ϕ̅*_*t*_⟩ in the zeroth-order description of the singlet wave function and derive a new set of working equations. Since the perturbational treatment is only directed at the individual exchange couplings *J*_tu_, we will refer to this method as an exchange-dedicated perturbation theory.

## 2 Exchange-Dedicated Perturbation Theory

The second-order exchange-dedicated perturbation theory method (EDPT2) uses analytically derived equations to calculate the energy difference between high-spin and low-spin states of the same electronic configuration, i.e., the exchange interaction. In this section, we will discuss the theoretical foundations of the EDPT2 method and present its algorithm as implemented in ORCA.^24^

### 2.1 Theoretical Foundations

The discussion of the energy difference between high-spin and low-spin states of the same electronic configuration starts by introducing the Heisenberg–Dirac-van-Vleck Hamiltonian:

1 $${Ĥ}^{\mathrm{HDVV}}=\underset{t<u}{\sum }-J_{tu}{Ŝ}_{t}{Ŝ}_{u}$$

where the coupling of two spins located in the orbitals ϕ_*t*_ and ϕ_*u*_ is parametrized by the exchange interaction *J*_*tu*_. The Hamiltonian Ĥ^HDVV^ describes the interactions between neutral determinants. In the case of a two-electron-two-center system, we can define a neutral space spanned by the determinants |*ϕ_t_ϕ̅*_*u*_⟩ and |*ϕ_u_ϕ̅*_*t*_⟩, where a bar on top denotes a β spin in the corresponding orbital. Hence, for a two-electron-two-center system, we obtain the following Hamiltonian matrix:
2

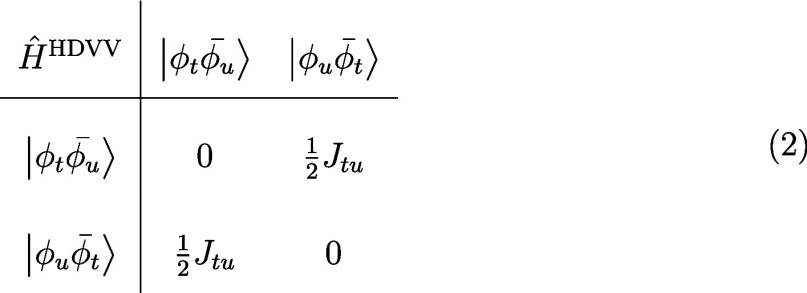




Diagonalization of this matrix yields a triplet wave function ψ_T_ and a singlet wave function ψ_S_:

3 $$\begin{array}{l} \psi_{T}=\frac{1}{\sqrt{2}}\left(\left|\phi_{t}{\mathrm{\phi ̅}}_{u}\right\rangle -\left|\phi_{u}{\mathrm{\phi ̅}}_{t}\right\rangle \right),\\ \psi_{S}=\frac{1}{\sqrt{2}}\left(\left|\phi_{t}{\mathrm{\phi ̅}}_{u}\right\rangle +\left|\phi_{u}{\mathrm{\phi ̅}}_{t}\right\rangle \right) \end{array}$$

which have an energy difference of:

4 $$\Delta E_{S\to T}=E_{S}-E_{T}=J_{\mathrm{tu}}$$

Replacing Ĥ^HDVV^ by the full Hamiltonian Ĥ leads to *J*_*tu*_ = 2 *K*_*tu*_ = 2(*tu*|*tu*), where we have used the chemist’s notation for two-electron integrals. However, this first-order description is incomplete since only the direct exchange is incorporated in *J*_*tu*_, which can only describe ferromagnetism.^1^

A better approach to the first-order description of *J*_*tu*_ is to incorporate the ionic configurations |*ϕ_u_ϕ̅*_*u*_⟩ and |*ϕ_u_ϕ̅_t_* ⟩ into the zeroth-order space. The corresponding Hamiltonian will take the following form:
5

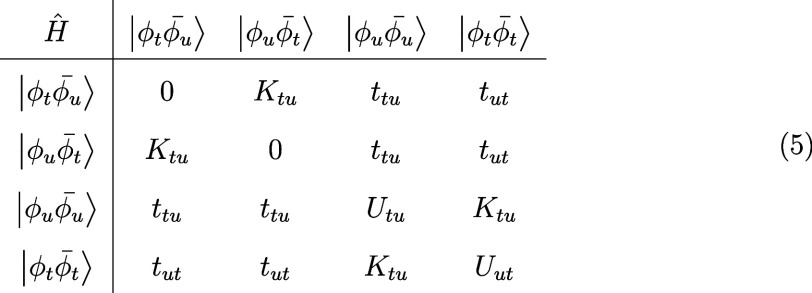




where we have introduced the hopping integrals *t*_*tu*_ and the one-site repulsion energies *U*_*tu*_.^5^ By introducing the core part of a CASSCF Fockian *f* ^core^_tu_:^18,19^

6 $$f_{tu}^{\mathrm{core}}=h_{tu}+\underset{i}{\sum }2\left(tu|ii\right)-\left(ti|iu\right)$$

where *h*_*tu*_ is an element of the one-electron matrix, we can define the hopping integrals *t*_*tu*_ and the one-site repulsion energies *U*_*tu*_ as

7 $$\begin{array}{ll} t_{tu} & =f_{tu}^{\mathrm{core}}+\left(tu|uu\right),\\ U_{tu} & =f_{uu}^{\mathrm{core}}-f_{tt}^{\mathrm{core}}+\left(uu|uu\right)-\left(tu|tu\right) \end{array}$$

The diagonalization of this model Hamiltonian will give a triplet state ψ^T^:

8 $$\psi^{T}=\frac{1}{\sqrt{2}}\left(\left|\phi_{t}{\mathrm{\phi ̅}}_{u}\right\rangle -\left|\phi_{u}{\mathrm{\phi ̅}}_{t}\right\rangle \right)$$

and three singlet states, whereby we are only interested in the singlet state $\psi_{N}^{S}$ , that has the largest projection onto the neutral subspace spanned by the neutral determinants |*ϕ_t_ϕ̅*_*u*_⟩ and |*ϕ_u_ϕ̅_t_* ⟩:

9 $$\psi_{N}^{S}=c_{N}\left(\left|\phi_{t}{\mathrm{\phi ̅}}_{u}\right\rangle +\left|\phi_{u}{\mathrm{\phi ̅}}_{t}\right\rangle \right)+c_{3}\left|\phi_{u}{\mathrm{\phi ̅}}_{u}\right\rangle +c_{4}\left|\phi_{t}{\mathrm{\phi ̅}}_{t}\right\rangle$$

with *c*_N_ ≫ *c*_3_ and *c*_N_ ≫ *c*_4_.^5^ The coefficients of the ionic states *c*_3_ and *c*_4_ are not necessarily equal, since ϕ_*t*_ and ϕ_*u*_ are not restricted to be degenerate. Now, the first-order exchange interaction $J_{tu}^{\left(0-1\right)}$ can be calculated by the energy difference of ψ^T^ and $\psi_{N}^{S}$ :

10 $$J_{tu}^{\left(0-1\right)}=E\left(\psi_{N}^{S}\right)-E\left(\psi^{T}\right)$$

which can be positive (ferromagnetic) or negative (antiferromagnetic) since the antiferromagnetic kinetic exchange is intrinsically incorporated. By means of quasi-degenerate perturbation theory, $J_{tu}^{\left(0-1\right)}$ can be rationalized as

11 $$J_{tu}^{\left(0-1\right)}=2K_{tu}-\frac{2t_{tu}^{2}}{U_{tu}}-\frac{2t_{ut}^{2}}{U_{ut}}$$

where the last two terms are known as the kinetic exchange contributions.^1^

At this level of theory, the exchange interaction *J*_*tu*_ is usually underestimated.^8,11,12,16,25,26^ However, the *J* value can be improved by introducing a second-order correction to the energies of ψ^T^ and $\psi_{N}^{S}$ :^16^

12 $$\begin{array}{l} E_{T}^{\left(0-2\right)}=\left\langle \psi^{T}\right|Ĥ\left|\psi^{T}\right\rangle +\underset{\mu r}{\sum }\frac{\left\langle \psi^{T}\right|Ĥ|{\psi_{\mu }^{r}\rangle }^{2}}{\left\langle \psi^{T}\right|{Ĥ}^{\left(0\right)}\left|\psi^{T}\right\rangle -\left\langle \psi_{\mu }^{r}\right|{Ĥ}^{\left(0\right)}\left|\psi_{\mu }^{r}\right\rangle }\\ E_{S}^{\left(0-2\right)}=\left\langle \psi_{N}^{S}\right|Ĥ\left|\psi_{N}^{S}\right\rangle +\underset{\mu r}{\sum }\frac{\left\langle \psi_{N}^{S}\right|Ĥ|{\psi_{\mu }^{r}\rangle }^{2}}{\left\langle \psi_{N}^{S}\right|{Ĥ}^{\left(0\right)}\left|\psi_{N}^{S}\right\rangle -\left\langle \psi_{\mu }^{r}\right|{Ĥ}^{\left(0\right)}\left|\psi_{\mu }^{r}\right\rangle } \end{array}$$

with $\psi_{\mu }^{r}$ being the μ’th determinant of a specific perturber class *r* that spans the first-order interacting space (FOIS).

The choice of Ĥ^(0)^ influences the convergence of the perturbation expansion.^27^ In a preceding paper,^8^ we have chosen Ĥ^(0)^ to be the sum of the CASSCF Fock operators. However, in doing so, we had to introduce two-electron interactions in the denominator in cases of charge polarization within the active space, which is formally not correct. This problem can be circumvented by using Dyall’s Hamiltonian^27^ as H^(0)^, which still uses a monoelectronic description of the inactive electrons but employs the full Hamiltonian within the active space:

13 $${Ĥ}^{\left(0\right)}={Ĥ}^{\mathrm{inact}}+{Ĥ}^{\mathrm{act}}$$

with Ĥ^inact^ acting on the inactive space:

14 $${Ĥ}^{\mathrm{inact}}=\underset{ij}{\sum }f_{ij}{Ê}_{ij}+\underset{ab}{\sum }f_{ab}{Ê}_{ab}+C$$

and Ĥ^act^ acting on the active space:

15 $${Ĥ}^{\mathrm{act}}=\underset{tu}{\sum }h_{tu}^{\mathrm{eff}}{Ê}_{tu}+\frac{1}{2}\underset{tuvx}{\sum }\left(tu|vx\right)\left({Ê}_{tu}{Ê}_{vx}-\delta_{uv}{Ê}_{tx}\right)$$

*f*_*ij*_ and *f*_*ab*_ are CASSCF Fock matrix elements:

16 $$f_{pq}=f_{pq}^{\mathrm{core}}+f_{pq}^{\mathrm{act}}$$

with:

17 $$f_{pq}^{\mathrm{core}}=h_{pq}+\underset{i}{\sum }2\left(pq|ii\right)-\left(pi|iq\right)$$

18 $$f_{pq}^{\mathrm{act}}=D_{tu}\underset{tu}{\sum }\left(pq|tu\right)-\frac{1}{2}\left(pt|uq\right)$$

where *D*_*tu*_ is an element of the first-order density matrix. The constant *C* ensures that Ĥ^(0)^ is equal to the full Hamiltonian projected onto the complete active space. The effective one-electron term $h_{tu}^{\mathrm{eff}}$ is defined as

19 $$h_{tu}^{\mathrm{eff}}=h_{tu}+\underset{i}{\sum }2\left(tu|ii\right)-\left(ti|iu\right)$$

which corresponds to the core part of the CASSCF Fockian $f_{tu}^{\mathrm{core}}$ .

The zeroth-order energies of ψ^T^ and $\psi_{N}^{S}$ can be assumed to be very close to each other compared to the energies of the perturbers $\psi_{\mu }^{r}$ . Thus, the denominator of the second-order terms can be simplified by using the energy of the neutral determinants |*ϕ_t_ϕ̅*_*u*_⟩ and |*ϕ_u_ϕ̅*_*u*_⟩ instead:

20 $$\left\langle \psi^{T}\right|{Ĥ}^{\left(0\right)}\left|\psi^{T}\right\rangle \approx \left\langle \psi_{N}^{S}\right|{Ĥ}^{\left(0\right)}\left|\psi_{N}^{S}\right\rangle \approx \left\langle \phi_{t}{\mathrm{\phi ̅}}_{u}\right|{Ĥ}^{\left(0\right)}\left|\phi_{t}{\mathrm{\phi ̅}}_{u}\right\rangle =\left\langle \phi_{u}{\mathrm{\phi ̅}}_{t}\right|{Ĥ}^{\left(0\right)}\left|\phi_{u}{\mathrm{\phi ̅}}_{t}\right\rangle$$

The second-order *J* value can therefore be written as

21 $$J_{tu}^{\left(0-2\right)}=E_{S}^{\left(0-2\right)}-E_{T}^{\left(0-2\right)}=J_{tu}^{\left(0-1\right)}+J_{tu}^{\left(2\right)}$$

where $J_{tu}^{\left(2\right)}$ is the second-order contribution to the exchange interaction:

22 $$J_{tu}^{\left(2\right)}=\underset{\mu r}{\sum }\frac{\left\langle \psi_{N}^{S}\right|Ĥ\left|{\psi_{\mu }^{r}\rangle }^{2}-\left\langle \psi^{T}\right|Ĥ\right|{\psi_{\mu }^{r}\rangle }^{2}}{\langle \phi_{t}{\mathrm{\phi ̅}}_{u}\left|{Ĥ}^{\left(0\right)}\right|\phi_{t}{\mathrm{\phi ̅}}_{u}\rangle -\left\langle \psi_{\mu }^{r}\right|{Ĥ}^{\left(0\right)}\left|\psi_{\mu }^{r}\right\rangle }$$

By inserting the wave functions (eqs 8 and 9) and rearranging $J_{tu}^{\left(2\right)}$ , we can decompose $J_{tu}^{\left(2\right)}$ into a neutral component $J_{tu,\mathrm{neutral}}^{\left(2\right)}$ and an ionic component $J_{tu,\mathrm{ionic}}^{\left(2\right)}$ :

$$J_{tu,\mathrm{neutral}}^{\left(2\right)}=\left(2c_{N}^{2}+1\right)\underset{r\mu }{\sum }\frac{\left\langle \phi_{t}{\mathrm{\phi ̅}}_{u}\right|Ĥ\left|\psi_{\mu }^{r}\right\rangle \langle \psi_{\mu }^{r}\left|Ĥ\right|\phi_{u}{\mathrm{\phi ̅}}_{t}\rangle }{\left\langle \phi_{t}{\mathrm{\phi ̅}}_{u}\right|{Ĥ}^{\left(0\right)}\left|\phi_{t}{\mathrm{\phi ̅}}_{u}\right\rangle -\left\langle \psi_{\mu }^{r}\right|{Ĥ}^{\left(0\right)}\left|\psi_{\mu }^{r}\right\rangle }$$ 23

$$J_{tu,\mathrm{ionic}}^{\left(2\right)}=2c_{N}\underset{\mu r}{\sum }\frac{\left(\langle \phi_{t}{\mathrm{\phi ̅}}_{u}|Ĥ\left|\psi_{\mu }^{r}\right\rangle +\langle \phi_{u}{\mathrm{\phi ̅}}_{t}|Ĥ\left|\psi_{\mu }^{r}\right\rangle \right)\left(c_{3}\langle \phi_{u}{\mathrm{\phi ̅}}_{u}|Ĥ\left|\psi_{\mu }^{r}\right\rangle +c_{4}\langle \phi_{t}{\mathrm{\phi ̅}}_{t}|Ĥ\left|\psi_{\mu }^{r}\right\rangle \right)}{\left\langle \phi_{t}{\mathrm{\phi ̅}}_{u}\right|{Ĥ}^{\left(0\right)}\left|\phi_{t}{\mathrm{\phi ̅}}_{u}\right\rangle -\left\langle \psi_{\mu }^{r}\right|{Ĥ}^{\left(0\right)}\left|\psi_{\mu }^{r}\right\rangle }+\underset{\mu r}{\sum }\frac{2c_{3}c_{4}\left\langle \phi_{u}{\mathrm{\phi ̅}}_{u}\right|Ĥ\left|\psi_{\mu }^{r}\right\rangle \left\langle \phi_{t}{\mathrm{\phi ̅}}_{t}\right|Ĥ\left|e\psi_{\mu }^{r}\right\rangle }{\left\langle \phi_{t}{\mathrm{\phi ̅}}_{u}\right|{Ĥ}^{\left(0\right)}\left|\phi_{t}{\mathrm{\phi ̅}}_{u}\right\rangle -\left\langle \psi_{\mu }^{r}\right|{Ĥ}^{\left(0\right)}\left|\psi_{\mu }^{r}\right\rangle }+\underset{\mu r}{\sum }\frac{c_{3}^{2}\left\langle \phi_{u}{\mathrm{\phi ̅}}_{u}\right|Ĥ|{\psi_{\mu }^{r}\rangle }^{2}}{\left\langle \phi_{t}{\mathrm{\phi ̅}}_{u}\right|{Ĥ}^{\left(0\right)}\left|\phi_{t}{\mathrm{\phi ̅}}_{u}\right\rangle -\left\langle \psi_{\mu }^{r}\right|{Ĥ}^{\left(0\right)}\left|\psi_{\mu }^{r}\right\rangle }+\underset{\mu r}{\sum }\frac{c_{4}^{2}\left\langle \phi_{t}{\mathrm{\phi ̅}}_{t}\right|Ĥ|{\psi_{\mu }^{r}\rangle }^{2}}{\left\langle \phi_{t}{\mathrm{\phi ̅}}_{u}\right|{Ĥ}^{\left(0\right)}\left|\phi_{t}{\mathrm{\phi ̅}}_{u}\right\rangle -\left\langle \psi_{\mu }^{r}\right|{Ĥ}^{\left(0\right)}\left|\psi_{\mu }^{r}\right\rangle }-\underset{\mu r}{\sum }\frac{\frac{1}{2}\left(c_{3}^{2}+c_{4}^{2}\right)\left(\langle \phi_{t}{\mathrm{\phi ̅}}_{u}|Ĥ|{\psi_{\mu }^{r}\rangle }^{2}+\langle \phi_{u}{\mathrm{\phi ̅}}_{t}|Ĥ|{\psi_{\mu }^{r}\rangle }^{2}\right)}{\left\langle \phi_{t}{\mathrm{\phi ̅}}_{u}\right|{Ĥ}^{\left(0\right)}\left|\phi_{t}{\mathrm{\phi ̅}}_{u}\right\rangle -\left\langle \psi_{\mu }^{r}\right|{Ĥ}^{\left(0\right)}\left|\psi_{\mu }^{r}\right\rangle }$$ 24

with:

25 $$J_{tu}^{\left(2\right)}=J_{tu,\mathrm{neutral}}^{\left(2\right)}+J_{tu,\mathrm{ionic}}^{\left(2\right)}$$

In case of a totally neutral singlet state (i.e., $c_{N}=\frac{1}{\sqrt{2}}$ ), $J_{tu}^{\left(2\right)}$ reduces to the expression obtained by perturbing only the direct exchange *K*_*tu*_ with the use of quasi-degenerate perturbation theory, as it can be readily seen in the expression of $J_{tu,\mathrm{neutral}}^{\left(2\right)}$ .^1,28,29^ The ionic component of the second-order correction $J_{tu,\mathrm{ionic}}^{\left(2\right)}$ arises solely from the presence of ionic amplitudes in the singlet wave function $\psi_{N}^{S}$ and can therefore be seen as a correction to the bare kinetic exchange.

To derive the working equations of the EDPT2 method, we classify the perturbers ψ^*r*^ into nine different groups:

- 1h configurations
- 1p configurations
- 1h-1p configurations → single excitations (SE)
- 1h-1p configurations → dynamic spin polarization (DSP)
- 1h-1p configurations → dynamic charge polarization (DCP)
- 2h configurations
- 2p configurations
- 2h-1p configurations
- 1h-2p configurations

An ″h″ corresponds to a hole in the internal space, while a ″p″ corresponds to a created particle in the virtual space. These configurations contribute to the exchange interaction at the second order.^16,30,31^ Figure 1 shows the basic occupation schemes for the corresponding perturber classes ψ^*r*^.

**Figure 1:**
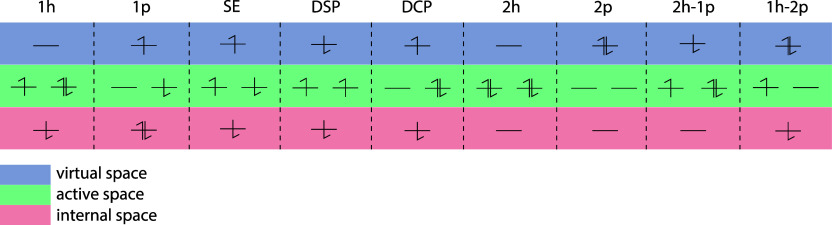
Basic occupation schemes for the perturber classes in the EDPT2 method. The single excitations (SE), as well as the 2h-1p and 1h-2p classes, only influence the ionic component of *J*_*tu*_, while the remaining perturber classes influence both the neutral component and the ionic component of *J*_*tu*_.

The explicit equations for the effects of the above configurations on the exchange interaction *J*_*tu*_ can be found in the Supporting Information. However, we will still demonstrate the derivation of the working equations, taking the 2p configurations as an example. Here, the FOIS is spanned by determinants of the type |*ϕ_a_ϕ̅*_*b*_⟩, whereby *a* and *b* run over the whole virtual space. Now, we need to derive four distinct integrals occurring in the numerators and two distinct integrals occurring in the denominator. This is carried out with the use of the Slater–Condon rules:

26 $$\begin{array}{l} \left\langle \phi_{t}{\mathrm{\phi ̅}}_{u}\right|Ĥ\left|\phi_{a}{\mathrm{\phi ̅}}_{b}\right\rangle =\left(ta|ub\right)\\ \left\langle \phi_{u}{\mathrm{\phi ̅}}_{t}\right|Ĥ\left|\phi_{a}{\mathrm{\phi ̅}}_{b}\right\rangle =\left(ua|tb\right)\\ \left\langle \phi_{u}{\mathrm{\phi ̅}}_{u}\right|Ĥ\left|\phi_{a}{\mathrm{\phi ̅}}_{b}\right\rangle =\left(ua|ub\right)\\ \left\langle \phi_{t}{\mathrm{\phi ̅}}_{t}\right|Ĥ\left|\phi_{a}{\mathrm{\phi ̅}}_{b}\right\rangle =\left(ta|tb\right)\\ \left\langle \phi_{t}{\mathrm{\phi ̅}}_{u}\right|{Ĥ}^{\left(0\right)}\left|\phi_{t}{\mathrm{\phi ̅}}_{u}\right\rangle -\left\langle \phi_{a}{\mathrm{\phi ̅}}_{b}\right|{Ĥ}^{\left(0\right)}\left|\phi_{a}{\mathrm{\phi ̅}}_{b}\right\rangle =f_{tt}^{\mathrm{core}}+f_{uu}^{\mathrm{core}}-\epsilon_{a}-\epsilon_{b}+\left(tt|uu\right) \end{array}$$

Inserting these integrals into $J_{tu,\mathrm{neutral}}^{\left(2\right)}$ and $J_{tu,\mathrm{ionic}}^{\left(2\right)}$ , we obtain

27 $$J_{tu,\mathrm{neutral},2p}^{\left(2\right)}=\left(2c_{N}^{2}+1\right)\underset{ab}{\sum }\frac{\left(ta|ub\right)\left(ua|tb\right)}{f_{tt}^{\mathrm{core}}+f_{uu}^{\mathrm{core}}-\epsilon_{a}-\epsilon_{b}+\left(tt|uu\right)}$$

28 $$J_{tu,\mathrm{ionic},2p}^{\left(2\right)}=2c_{N}\underset{ab}{\sum }\frac{\left(\left(ta|ub\right)+\left(ua|tb\right)\right)\left(c_{3}\left(ua|ub\right)+c_{4}\left(ta|tb\right)\right)}{f_{tt}^{\mathrm{core}}+f_{uu}^{\mathrm{core}}-\epsilon_{a}-\epsilon_{b}+\left(tt|uu\right)}+\underset{ab}{\sum }\frac{2c_{3}c_{4}\left(ua|ub\right)\left(ta|tb\right)+c_{3}^{2}{\left(ua|ub\right)}^{2}+c_{4}^{2}{\left(ta|tb\right)}^{2}}{f_{tt}^{\mathrm{core}}+f_{uu}^{\mathrm{core}}-\epsilon_{a}-\epsilon_{b}+\left(tt|uu\right)}-\frac{1}{2}\underset{ab}{\sum }\frac{\left(c_{3}^{2}+c_{4}^{2}\right)\left({\left(ta|ub\right)}^{2}+{\left(ua|tb\right)}^{2}\right)}{f_{tt}^{\mathrm{core}}+f_{uu}^{\mathrm{core}}-\epsilon_{a}-\epsilon_{b}+\left(tt|uu\right)}$$

After computing the transformed integrals and the coefficients *c*_N_, *c*_3_ and *c*_4_, the second-order contributions to the exchange interaction can be calculated directly using analytically derived expressions, as demonstrated for the 2p configurations.

Although the working equations are derived for two-spin systems, the EDPT2 method can also be used for multispin systems. For example, in the case of a three-spin system, the EDPT2 method calculates the three distinct exchange interactions *J*_*tu*_, *J*_*tv*_, and *J*_*uv*_, which can be inserted into a Heisenberg Hamiltonian matrix. After diagonalization of this matrix, one obtains the relative energies of a quartet state and two doublet states, which are only composed of neutral determinants.^7,32^ Regarding the description of multispin systems with more than two spins, it should be noted that only those corrections to the exchange integrals that arise from possible single and double excitations within the active space are considered. Details regarding these additional contributions can be found in the Supporting Information.

In cases where the reference wave functions are obtained from an active space that is larger than the corresponding minimal active space of a given magnetic system (two spin centers → CASSCF­(2,2), three spin centers → CASSCF­(3,3), and so on), the EDPT2 method may break down since it is derived for the case of dominating amplitudes of the neutral configurations. This is not the case when using general methods such as, for example, the *n*-electron valence state perturbation theory^33–35^ (NEVPT2) or the complete active space perturbation theory^36,37^ (CASPT2).

Another distinction between the EDPT2 method and the NEVPT2 or CASPT2 methods lies in the treatment of the first-order interacting space. EDPT2 employs an externally decontracted approach, meaning the perturbers $\langle \psi_{\mu }^{r}$ are individual Slater determinants. In contrast, NEVPT2 and CASPT2 use a contracted description, where perturbers are generated by applying excitation operators to the entire zeroth-order wave function rather than to individual determinants. This determinant-level resolution in EDPT2 offers greater flexibility for describing dynamical correlation, albeit at a higher computational cost.^1,38^

Despite this distinction, all three methods – EDPT2, NEVPT2, and CASPT2 – are classified as internally contracted. That is, the zeroth-order wave functions remain fixed and are not variationally relaxed in response to the first-order interacting space. As a result, the first-order corrected wave function takes the form

$$|\psi_{0}^{\left(0-1\right)}\,\rangle =|\psi_{0}^{\left(0\right)}\,\rangle +\underset{i}{\sum }c_{i,0}\phi_{i}$$

where the ϕ_*i*_ functions lie outside the zeroth-order (reference) space. This restriction can impact properties such as magnetic exchange couplings, since the ratio between neutral and ionic reference determinants is not modified by dynamical correlation in this formulation.^38^

Extensions of quasi-degenerate perturbation theory exist for both NEVPT2 and CASPT2, allowing partial relaxation of the zeroth-order wave functions.^34,39^ Furthermore, Angeli and coworkers have proposed a numerical state-specific decontraction scheme, in which the reference wave function is perturbed slightly to account for decontraction effects in a more flexible but computationally efficient manner.^38^

### 2.2 Details on the Algorithm

The EDPT2 method requires an initial CASCI­(*n*,*n*) calculation, after which it calculates an *n* × *n* matrix with the second-order corrected exchange interactions as off-diagonal elements of that matrix. In the case of an initial CASCI­(2,2) calculation, the EDPT2 method will produce a matrix of the form:

29 $$\left(\begin{array}{cc} 0 & J_{12}^{\left(0-2\right)}\\ J_{12}^{\left(0-2\right)} & 0 \end{array}\right)$$

(1) In the first step, the active orbitals are localized,^40^ as this is required to describe the low-lying high-spin and low-spin states by means of neutral determinants.

(2) Unlike the previous implementation,^8^ which generated a (diagonal) CASSCF Fock matrix in the basis of localized orbitals at this stage, the current approach uses the exact CASSCF Fock matrix (*f*_CASSCF_= $f_{\mathrm{CASSCF}}^{\mathrm{core}}+f_{\mathrm{CASSCF}}^{\mathrm{act}}$ ) in the localized orbital basis. The core component of the CASSCF Fock matrix is essential for computing the single-excitation terms as well as the zeroth-order energies accurately. Consequently, this implementation accounts for the nonzero contributions of the orbital relaxation terms (1h and 1p contributions), which were neglected in the earlier implementation.

(3) At this stage, the two-electron integrals are transformed in the localized orbital basis. In contrast to the previous implementation, where (*ik*| *jl*) and (*ia*| *jb*) integrals were generated, only those integrals are generated which are essential for the calculation of the effective exchange integrals. This involves the generation of integrals of the following types: (*tu*| *ia*), (*it*| *uj*), (*it*| *ua*), (*at*| *ub*), (*it*| *ja*), and (*ta*| *ib*). The generation of these integrals is carried out faster than the generation of the more numerous (*ia*| *jb*) integrals.

(4) For each pair of active indices, the 4 × 4 Hamiltonian (eq 5) is constructed and diagonalized in order to obtain the coefficients *c*_N_, *c*_3_ and *c*_4_, as well as $J_{tu}^{\left(0-1\right)}$ . The coefficients *c*_N_, *c*_3_ and *c*_4_ belong to the singlet state $\psi_{N}^{S}$ that has the largest norms on the neutral determinants |ϕ_*t*_ϕ̅_*u*_⟩ and |ϕ_*u*_ϕ̅_*t*_⟩. $J_{tu}^{\left(0-1\right)}$ is then computed as the energy difference of that singlet state and the triplet state.

(5) In the final step, for each pair of active indices, the second-order contributions to the exchange interactions $J_{tu}^{\left(2\right)}$ are computed by using the analytically derived expressions shown in the Supporting Information. Finally, the exchange interactions *J*_*tu*_ are expressed in matrix form.

At present, the EDPT2 method is implemented as a pilot code. Full integration into the ORCA program package is planned for the near future.

## 3 Results and Discussion

### 3.1 Choice of Molecules

The EDPT2 method is evaluated using 10 test systems, where singlet–triplet splittings are calculated. The results of the EDPT2 method are compared to those of the FIC-NEVPT2 method, as well as with experimental values from the literature.^41–48^ The FIC-NEVPT2 method serves as a suitable reference method since it is also a second-order multireference perturbation theory method that employs the partially bielectronic Dyall Hamiltonian as the zeroth-order Hamiltonian.

The test set consists of (1) methylene, the aromatic systems (2) benzene, (3) naphthalene, (4) anthracene, and (5) tetracene, as well as four additional nitroxide biradicals based on (6) benzene (para-substituted), (7) benzene (meta-substituted), (8) pyrene, (9) 1,1’-biphenyl, and (10) benzo­[1,2-b:4,5-b’]­dithiophene. The corresponding structures are shown in Figure 2.

**Figure 2:**
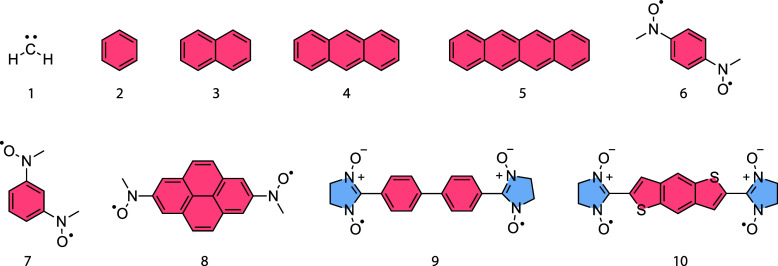
Test set consists of ten molecules comprising (1) methylene, (2–5) basic aromatic compounds, and (6–10) nitroxide biradicals. The singlet–triplet gaps of these compounds were calculated using the EDPT2 and the FIC-NEVPT2 methods.

### 3.2 Computational Details

All calculations were carried out using a developer version of ORCA, which corresponds to ORCA 6.0.^24^ The (triplet state) structures were optimized at the B3LYP/6–311G* level of theory.^49–53^ The optimizations were accelerated using the RIJCOSX approximation^54^ in conjunction with the def2/J auxiliary function.^55^ Prior to the EDPT2 and the FIC-NEVPT2 calculations, CASSCF­(2,2) calculations were carried out using the 6–311G* basis set, whereby the orbitals were optimized to the triplet state. Again, the RIJCOSX approximation was employed using the def2/J auxiliary basis. The integral transformations were accelerated using the resolution of the identity (RI) approximation with the def2-SVP/C auxiliary basis.

### 3.3 Scaling of the EDPT2 Method

The most expensive step of the current EDPT2 implementation is the construction of the (*ta*|*ib*) and (*it*|*ja*) integrals:^56^

30 $$\left(ta|ib\right)=\underset{k}{\sum }\left(ta|k\right)\left(k|ib\right)$$

31 $$\left(it|ja\right)=\underset{k}{\sum }\left(it|k\right)\left(k|ja\right)$$

where *k* is an auxiliary function used within the resolution of the identity approximation. Thus, the scaling of the EDPT2 method becomes

32 $$t=m\cdot \left(N_{k}N_{\mathrm{act}}N_{\mathrm{int}}N_{\mathrm{virt}}^{2}+N_{k}N_{\mathrm{act}}N_{\mathrm{int}}^{2}N_{\mathrm{virt}}\right)$$

with *N*_*k*_ being the number of auxiliary functions and *N*_act_, *N*_int_ and *N*_virt_ being the number of active, internal, and virtual orbitals, respectively. While *N*_act_ is independent of the number of basis functions *N*, *N*_*k*_ can be rewritten as a function of *N*:

33 $$N_{k}=xN$$

Thus, the scaling becomes proportional to

34 $$t\propto N\left(N_{\mathrm{int}}N_{\mathrm{virt}}^{2}+N_{\mathrm{int}}^{2}N_{\mathrm{virt}}\right)$$

Using the relation *N* = *N*_int_+*N*_virt_, (eq 34) can be written as

35 $$t\propto N^{2}N_{\mathrm{int}}N_{\mathrm{virt}}$$

While the formal scaling of the RI-integral transformation is *N* ^4^ (with a small prefactor), in practice, we observe a scaling that is as favorable as *N* ^3^ and *N* ^4^ depending on the ratio *N*_int_/*N*_virt_ and the basis set size, which is in line with the fitted scaling curve in Figure 3. This is a key advantage over NEVPT2 that scales as *N* ^5^ independent of whether the RI approximation is used or not.

**Figure 3:**
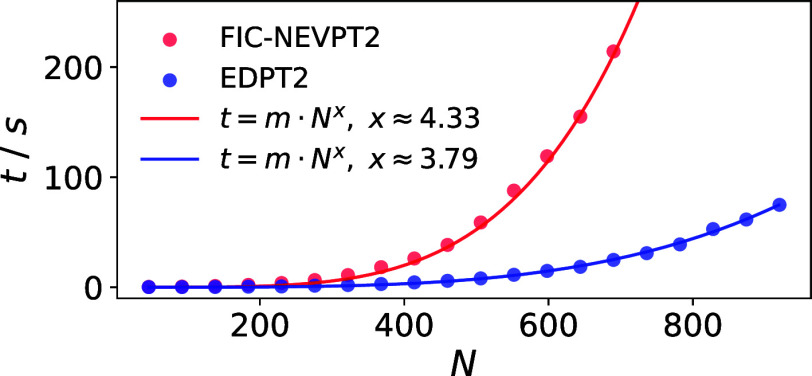
Scaling of the EDPT2 method and the FIC-NEVPT2 method with the basis set size *N*. The blue and red dots represent the actual timings using the EDPT2 method and the NEVPT2 method, respectively. The NEVPT2 and EDPT2 timings are fitted using a fit function of the type *mN* ^*x*^ (blue and red lines). It is important to note that both methods were used in conjunction with the RI-approximation. The calculations were carried out on different numbers of Xe atoms using the def2-TZVP basis set.

### 3.4 Benchmark Results

As can be seen from Table 1, the calculated singlet–triplet splittings, or simply *J*, generally compare well with the experimental values. The sign of the calculated splittings is always predicted correctly, and the magnitude of the calculated values is in line with the experimental values. Larger deviations are observed for compounds **1**, **7**, and **8**. In the case of methylene **1**, the large deviation is likely due to the fact that the triplet-optimized structure, as well as triplet-optimized orbitals, were used for the calculation of *J*. Such a procedure may lead to a bias toward the triplet state, as observed here for **1**. This issue is less severe for the remaining compounds, as only minor structural changes may occur by using state-specific structures. In addition, the molecular orbitals may only show minor differences in the case of state-specific orbital optimizations. In the case of compounds **7** and **8**, the larger deviations might be rooted in the type of radical employed. The orbitals localized on the nitroxides might need to be more diffuse in order to account for the correct magnitude of *J*, as we already demonstrated in a previous study.^8^ This can be achieved by using state-averaging in the CASSCF procedure, where energetically higher lying (low-spin) states are considered (to some extent) in the orbital optimization.^8,25,57^

**Table 1:** Calculated and Experimental Singlet–Triplet Splittings of the Compounds **1**–10^a^,^b^

	EDPT2	FIC-NEVPT2	Exp.
**1**	10,176	9384	3165
**2**	–31,126	–28,783	–29,500
**3**	–21,546	–19,096	–21,180
**4**	–12,967	–12,420	–14,870
**5**	–9771	–8905	–10,250
**6**	–885	–885	-
**7**	198	185	695
**8**	–128	–128	–823
**9**	–16.3	–16.3	–19.5
**10**	–51.5	–51.5	–36

a All values are given in cm^–1^.

b The orbitals were generated using CASSCF­(2,2), whereby the orbitals were optimized towards the triplet state.

When examining the calculated singlet–triplet splittings for the basic aromatic compounds **2**–**5**, it can be seen that they compare particularly well with the experimental values. This shows that the EDPT2 method is not necessarily restricted to magnetic problems, but can be used in general to calculate low-spin/high-spin gaps as long as the target states are mainly described by neutral determinants. The EDPT2 and FIC-NEVPT2 values compare very well with each other, whereby the calculated *J* values for the compounds **6**, **8**, **9**, and **10**, using the EDPT2 or FIC-NEVPT2 method, are practically identical.

A possible reason for the slight deviations in the calculated *J* values using the EDPT2 and NEVPT2 methods for compounds **1**–**5** may, to some extent, lie in the approximation expressed in eq 20. In this approach, the zeroth-order energies of the triplet and predominantly singlet states are approximated by the expectation values of the energy of neutral determinants within the model space. This approximation can introduce errors, particularly when there is a substantial energy gap between the singlet and triplet states, especially in systems where low-lying excited states lie close to the model space.

The validity of the approximation in eq 20 can be assessed by examining the direct exchange term *K*_*tu*_ and the first-order exchange interaction $J_{tu}^{\left(0-1\right)}$ . For instance, in the methylene biradical **1**, we find a first-order exchange splitting of $J_{tu}^{\left(0-1\right)}=12171{\mathrm{cm}}^{-1}$ and a direct exchange value of *K*_*tu*_ = 8025 cm^–1^. As a result, the triplet state energy is effectively raised by 8025 cm^–1^, while the singlet state is lowered by 4145 cm^–1^ under the eq 20) approximation. Although these shifts may initially appear significant, they become relatively minor when considered in the context of the energy separation between active-space and outer-space orbitals. For example, if we approximate the excitation energy of the lowest-lying 1h configuration by the difference in orbital energies, we obtain ⟨*ϕ*_*t*_ *ϕ̅*_*u*_|Ĥ^(0)^|*ϕ*_*t*_ *ϕ̅*_*u*_⟩ - ⟨*ϕ*_*t*–1_ *ϕ̅*_*t*_ *ϕtϕ̅*_*u*_|Ĥ^(0)^|*ϕ*_*t*_ *_–1_ϕ̅_t_ϕ_t_ϕ̅*_*u*_⟩ = 109376cm^–1^, suggesting that the error introduced by the approximation is comparatively small.

Consequently, the EDPT2 method can be used as an efficient alternative to common MRPT2 methods when describing the low-lying neutral states by means of minimal active space reference wave functions.

### 3.5 Class-Partitioned MRCI Calculations on the Magnetic Compounds

As can be seen in Table 1, the calculated singlet–triplet splittings for the magnetic compounds seem to deviate more strongly from the experimental values, especially for compounds **7**, **8**, and **10**. The reason for the discrepancy between the calculated and experimental values is the absence of higher-order interactions.^5,11,58^ Class-partitioned multireference configuration interaction (MRCI) calculations, where selective CI spaces are diagonalized, are a very useful tool for the identification of important interactions and will therefore be used here to identify the possibly missing higher-order interactions for these compounds in MRPT2 methods. Table 2 lists the calculated *J* values for compounds **6**–**10** using various CI spaces. Here, the largest CI space is defined by the DDCI3 space (difference-dedicated configuration interaction – with three degrees of freedom).^30,31,59^ The DDCI3 involves all single and double excitations except for the 2h-2p excitations, which do not have a differential effect on the eigenvalues within the CAS at the second order of perturbation. By leaving out the excitations with three degrees of freedom (2h-1p and 1h-2p) from the DDCI3 space, the DDCI2 space is obtained. The DDCI2 space can be further reduced to the DDCI1 space by leaving out the excitations with two degrees of freedom (2h and 2p). The DDCI methods are widely used for the calculation of exchange couplings.^5,58,60–72^

**Table 2:** Calculated *J* Couplings at the CASSCF­(2,2)+DDCI-*x*/6-31G* Level of Theory^a^ ^b^

	6	7	8	9	10
CASSCF(2,2)	–487	163	–64	0	0
CAS+2h–1p+1h	–771	386	–115	0.48	1.47
CAS+2h–1p+1h+1h–1p	–1679	571	–309	–4.15	–12.9
CAS+1h–1p	–1006	491	–191	–9.22	–27.7
CAS+1h–1p+1h	–1327	620	–254	–9.10	–27.2
CAS+1h–1p+1p	–1227	492	–221	–9.26	–27.6
DDCI1	–1724	632	–307	–9.13	–27.5
DDCI2	–1922	595	–327	–9.13	–27.4
DDCI2+2h–1p	–2218	548	–405	–4.36	–12.6
DDCI2+1h–2p	–1564	402	–455	–6.66	–20.5
DDCI3	–1785	375	–471	–2.62	–8.00
EDPT2	–885	198	–128	–16.3	–51.5
Exp.	-	695	–823	–19.5	–36

a Note that the EPT2 method was applied in conjunction with the 6-311G* basis set .

b In order to keep the DDCI3 calculations tractable, the energy window of orbitals to be included in the CI was set from −1.0 Eh to 1.0 Eh (except for compounds 6 and 7).

At the CASSCF­(2,2) level of theory, the calculated *J* values are underestimated for all compounds, which is typical for CASSCF calculations with a minimal active space.^5^

When extending the CAS to the DDCI1 space, the calculated *J* values are significantly improved for all compounds, highlighting the importance of the 1h-1p configurations in organic compounds. For compounds **6**–**8**, the effect of the 1h-1p configurations is amplified by both 1h and 1p configurations, while for compounds **9** and **10**, the DDCI1 *J* value is practically obtained by only considering a CAS+1h-1p CI space. In order to explain this behavior, one likely has to distinguish between DCP and DSP configurations within the 1h-1p configurations. As can be seen in Table 3, compounds **6**–**8** show important amplitudes for both DSP and DCP configurations, while compounds **9** and **10** show only a significant amplitude for the DSP configurations interacting directly with the neutral determinants of the model space. In fact, the DSP effect is practically the only relevant effect up to the second order of perturbation for these compounds.

**Table 3:** Specific Second-Order Contributions of 1h-1p Configurations to the Neutral and Ionic Part of the Exchange Coupling Calculated at the CASSCF­(2,2)+EDPT2/6-311G* Level of Theory

	6	7	8	9	10
$J_{neutral,DSP}^{\left(2\right)}$	–113	135	–29.0	–16.3	–51.5
$J_{\mathrm{ionic},\mathrm{DSP}}^{\left(2\right)}$	25.0	0.198	2.61	0	0
$J_{\mathrm{neutral},\mathrm{DCP}}^{\left(2\right)}$	–46.1	–49.8	–3.84	0	0
$J_{\mathrm{ionic},\mathrm{DCP}}^{\left(2\right)}$	–230	0.939	–25.5	0	0

The dynamic spin polarization effect, the importance of which was already described in detail,^5,9,12,16,73^ is characterized by the interaction of the spin-polarized neutral excited determinants (i.e., triplet polarization in the outer space and in the active space) with the neutral reference determinants. The mechanism of the dynamic spin polarization effect is illustrated in Figure 4. The magnitude of the effect depends on the coupling of the internal and virtual orbitals *i* and *a* by the local exchange operators K̂_*t*_ and K̂_*u*_ and becomes larger in the case of a strong overlap of the internal and virtual orbitals with the active orbitals, *t* and *u*.^16^ Consequently, the internal orbitals and the virtual orbitals have to act as bridging orbitals with low-lying triplet excitation energies.

**Figure 4:**
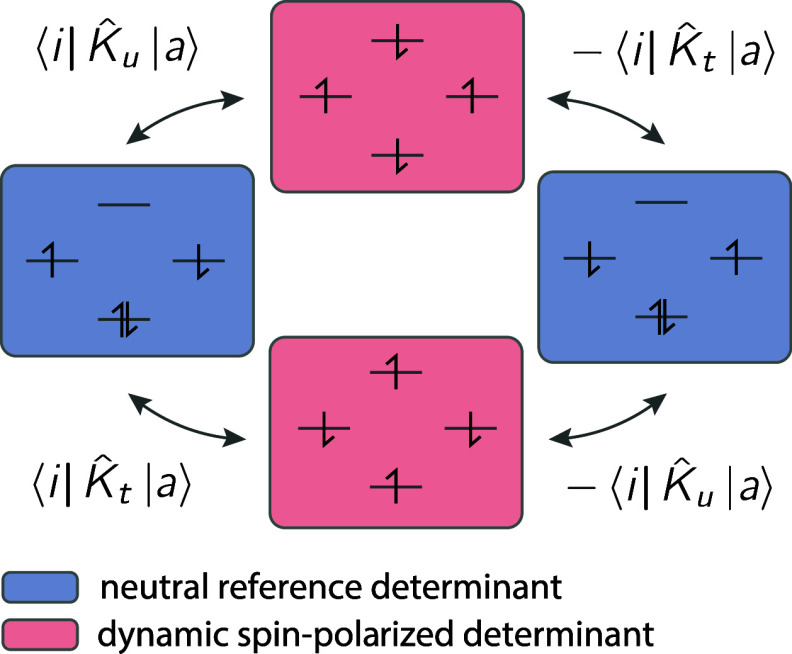
The main contribution to the exchange interaction for compounds **9** and **10** comes from the dynamic spin polarization effect, where the neutral reference configurations are coupled by doubly excited determinants that have a spin polarization in the active space and in the outer space. The coupling of the determinants is proportional to the coupling of the internal orbitals *i* and virtual orbitals *a* by the local exchange operators K̂_*t*_ and K̂_*u*_.

It is likely that at higher orders of perturbation (within the DDCI1 space), only the effect of the DCP configurations will be significantly influenced by the presence of the 1h and 1p configurations, while the 1h and 1p configurations will not interact strongly with the DSP configurations, as indicated by the CAS+1h-1p and CAS+1h-1p+1h entries.

The inclusion of the 2h and 2p configurations, leading to the DDCI2 space, only introduces a comparably small antiferromagnetic contribution to the exchange coupling for all compounds, as is also the case at the second-order of perturbation. For compounds **9** and **10**, the effect of the 2h and 2p configurations is negligible, indicating that the relevance of the 2h and 2p configurations in a CI calculation may be qualitatively measured by the relative contributions of the 2h and 2p configurations in MRPT2 calculations.

The introduction of the 2h-1p and 1h-2p configurations, leading to the DDCI3 space, yields a reduction of the DDCI2 value of the exchange interaction for all compounds except **8**. While for transition metal complexes the 2h-1p configurations increase the exchange coupling and the 1h-2p configurations dampen the exchange coupling, we do not observe such consistent behavior of the 2h-1p and 1h-2p configurations for these compounds.^11,12^ For compound **6**, the 2h-1p and 1h-2p configurations act in a similar way as for transition metal complexes. However, the dampening effect of the 1h-2p configurations outweighs the amplifying effect of the 2h-1p configurations. For compounds **7**, **9**, and **10**, the 2h-1p and 1h-2p configurations both decrease the exchange coupling with respect to the DDCI2 value, while for compound **8**, the 2h-1p and 1h-2p configurations both increase the exchange coupling. The diverse effects of the 2h-1p configurations become already visible in a (CAS+2h-1p+1h+1h-1p) CI treatment. When comparing the DDCI1, DDCI2, and DDCI3 values for the exchange interaction of these compounds with the experimental values, DDCI1 and DDCI2 seem to perform best, except for compound **8**, although none of the methods seem to predict the exchange coupling accurately. This behavior for organic compounds has already been observed in different studies,^12,74,75^ where the DDCI2 method predicted the exchange coupling more accurately than the DDCI1 and DDCI3 methods. As already stated by Calzado et al.,^12^ a model space containing only the neutral determinants of the minimal CAS could be assumed to be more physical than a minimal CAS for organic compounds, due to the fact that the ionic forms do not necessarily play an important role for these compounds. In such a case, the DDCI2 space is already complete with respect to all differential effects occurring at the second order. Including the 2h-1p and 1h-2p configurations in these cases, unphysical or incomplete effects are introduced, which may cancel out by including higher-order excitations not included in the DDCI3. To improve the accuracy, one could extend the active space by including more π orbitals (CAS­(6,6) in the case of the nitronyl nitroxides)^12^ or, as shown by Suaud et al.^58^ one may remain within a minimal CAS reference by using iteratively optimized natural orbitals from CAS+DDCI calculations restricted on the π manifold.

To improve the calculated *J* value, we applied a Davidson correction on top of the singlet and triplet energies, as was successfully demonstrated by Negodaev et al.^60^ As shown in Table 4, a Davidson correction applied on top of the DDCI energies yields *J* values that are very close to the experimental values. For compounds **7**, **8**, and probably **6**, the DDCI3 space has to be used in order to obtain *J* values within the experimental range, whereas the DDCI2 method yields an acceptable value for the ferromagnetic compound **7** compared to the antiferromagnetically coupled compounds **6** and **8**. Interestingly, for compounds **9** and **10**, the DDCI1 or, slightly better, the DDCI2 have to be used in order to obtain accurate *J* values when combined with the Davidson correction, whereas DDCI3 + Davidson correction fails to predict the *J* coupling. This indicates that compounds with dominating dynamic spin polarization may be best described by a neutral model space, as the DSP configurations do not interact strongly with the ionic forms (already indicated in Table 3). Furthermore, the DSP configurations cannot interact directly with the 2h-1p and 1h-2p configurations. For compounds with dominating kinetic exchange (antiferromagnetic coupling at the zeroth order) the DDCI3 space is more appropriate when combined with the Davidson correction.

**Table 4:** Calculated *J* Couplings at the CASSCF­(2,2)+DDCI-*x*/6-31G* Level of Theory using the Davidson Correction on the Energies of the Triplet and Singlet States^a^

	6	7	8	9	10
DDCI1	–2120	780	–395	–15.5	–33.5
DDCI2	–2391	739	–418	–15.8	–34.5
DDCI3	–3145	684	–823	–4.9	–11.8
EDPT2	–885	198	–128	–16.3	–51.5
Exp.	-	695	–823	–19.5	–36

a The EDPT2 method was applied in conjunction with the 6-311G* basis set.

Although the EDPT2 method does not necessarily predict exchange couplings within the experimental range, it can be used as a (second-order) analytical tool that helps to choose a reasonable CI space for selective MRCI calculations.

### 3.6 Three-Spin Systems: a Trisnitroxide Triradical

In this section, we demonstrate the application of the EDPT2 method on a three-spin system. As an example, we choose a trisnitroxide triradical shown in Figure 5. This compound has an experimentally determined doublet–quartet gap of △*E*_D1_→Q = 170cm^–1^.^45^ The experimental gap value was determined by fitting an approximate Heisenberg Hamiltonian only with nearest-neighbor interactions, i.e., Ĥ = −*J*(Ŝ_1_Ŝ_2_ + Ŝ_2_Ŝ_3_), where the centers 1 and 3 correspond to the terminal nitroxides, and center 2 corresponds to the internal nitroxide. In this case, the doublet–quartet splittings become △*E*_D1_→Q = *J*/2 and △*E*_D2→D1_ = *J*.

**Figure 5:**
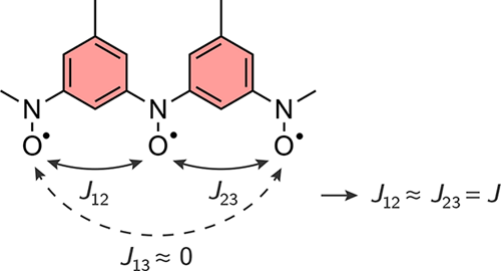
A linear trisnitroxide with phenyl bridges. The experimental doublet–quartet gap was measured to be △*E*_D1→Q_ = 170 cm^–1^. The exchange interaction between the terminal nitroxides is negligible due to the larger distance between the terminal nitroxide groups compared to the distance between neighboring nitroxides.

At the CASSCF­(3,3)/6–31G* level with optimization on the quartet state, a theoretical gap value of $E_{D_{1}\to Q}^{\mathrm{CASSCF}(3,3)}=40.2{\mathrm{cm}}^{-1}$ is obtained, which has the correct sign but underestimates the *J* coupling. When using the EDPT2 method subsequently, only a slightly improved value of $E_{D_{1}\to Q}^{\mathrm{EDPT}2}=51.9{\mathrm{cm}}^{-1}$ is obtained. By comparison, the NEVPT2 method yields a quite similar value of $E_{D_{1}\to Q}^{\mathrm{FIC}-\mathrm{NEVPT}2}=49.0{\mathrm{cm}}^{-1}$ .

$E_{D_{1}\to Q}^{\mathrm{EDPT}2}$ was obtained by diagonalizing the Heisenberg Hamiltonian (eq 1)) using the computed individual *J* couplings of the EDPT2 method: *J*_12_ = 106 cm^–1^, *J*_23_ = 106 cm^–1^, and *J*_13_ = −0.970 cm^–1^. Here, we can already see the validity of the approximate nearest-neighbor Heisenberg Hamiltonian for this compound, as *J*_13_ is negligibly small compared to the exchange interactions between the terminal nitroxides and the internal nitroxide, *J*_12_ and *J*_23_.

Analyzing the computed second-order contributions to the individual exchange couplings (*J*_12_ and *J*_23_), one finds two important second-order effects: the dynamic spin polarization $J_{\mathrm{DSP},\mathrm{neutral}}^{\left(2\right)}=71{\mathrm{cm}}^{-1}$ and the dynamic charge polarization effect $J_{\mathrm{DCP},\mathrm{neutral}}^{\left(2\right)}=-25{\mathrm{cm}}^{-1}$ with both effects originating from 1h-1p excited configurations. To some extent, the remaining second-order effects cancel out the contribution of the dynamic spin polarization.

Making use of the fact that the 1h-1p configurations represent the most important contributions at the second order and act almost exclusively on the neutral subspace of the CAS­(2,2), it may be reasonable to carry out a DDCI1 or, even better, a DDCI2 calculation. In the case of DDCI1, a strongly improved doublet–quartet gap of $E_{D_{1}\to Q}^{\mathrm{DDCI}1}=185{\mathrm{cm}}^{-1}$ is obtained, which is only slightly overestimated compared to the experimental value. Note that a Davidson correction is included in the gap value. If we include the EDPT2 contributions of the 2h and 2p configurations into the gap value (i.e., $J_{2h,\mathrm{neutral}}^{\left(2\right)}$ and $J_{2p,\mathrm{neutral}}^{\left(2\right)}$ ), we obtain a final theoretical value of *E*_D1→Q_ = 176cm^–1^, which compares well with the experimental value of 170 cm^–1^. The remaining second-order effects are negligible for the doublet–quartet gap. In the case of DDCI2 (again with the Davidson correction), a splitting of $E_{D_{1}\to Q}^{\mathrm{DDCI}2}=173{\mathrm{cm}}^{-1}$ is obtained, which is very close to the experimental value. The value of the DDCI1­(+EDPT2 corrections) compares well with that of the DDCI2 method.

## 4 Conclusion

In conclusion, we introduced a second-order exchange-dedicated perturbation theory to compute exchange couplings in magnetic systems and, consequently, their high-spin/low-spin gaps. The EDPT2 theory, which is firmly rooted in the pioneering work of Malrieu and coworkers,^16,17^ is derived on the two-electron-two-center case and can be used on multispin systems in general as long as a minimal CAS is used to generate the molecular orbitals. Due to the fact that EDPT2 uses a common orbital basis, the 2h-2p configurations do not need to be included in the perturbative treatment of the magnetic coupling, which leads to drastic computational savings. Thus, a very favorable scaling of *N* ^4^ is obtained.

We have shown that the EDPT2 method can be used to compute high-spin/low-spin gaps with comparable accuracy to the FIC-NEVPT2, and, presumably, other MRPT2 methods.

Besides the efficient scaling, the EDPT2 method has the advantage that all possible second-order contributions are calculated individually, which leads to an improved understanding of the important mechanisms behind the magnetic exchange. This was demonstrated on the nitronyl nitroxides **9** and **10**, where the dynamic spin polarization represents the only important exchange pathway at the second order and probably also at higher orders of perturbation. Furthermore, the insights gained from the EDPT2 method can be used to create more sophisticated calculation protocols for specific systems employing selected MRCI methods, as we have successfully demonstrated on a trisnitroxide triradical. Since the only important second-order contributions to the exchange couplings came from the dynamic spin-polarized and dynamic charge-polarized configurations acting almost exclusively on the neutral subspace of the CAS, a DDCI1 calculation (+Davidson correction) in combination with the 2h and 2p contributions from the EDPT2 method and a DDCI2 calculation (+Davidson correction) were carried out, yielding theoretical doublet–quartet gaps of Δ*E*_DDCI1+EDPT2_ = 176 cm^–1^ and Δ*E*_DDCI2_ = 173 cm^–1^ which match very well with each other and the experimental value of 170 cm^–1^.

While the EDPT2 method was benchmarked exclusively on organic systems, it is also applicable to polynuclear transition metal complexes, as long as a minimal CAS reference wave function is used.

Future work may focus on the generalization of this approach to the eigenstates of a CASCI, which would lead to a difference-dedicated multireference perturbation theory. It may also be interesting to derive an additional set of working equations that incorporate the relaxation of the dynamic spin-polarization effect and the dynamic charge-polarization effect through the 1h and 1p determinants, as the sole appearance of those perturber classes in a CI can lead to a drastic improvement of the calculated high-spin/low-spin gaps.

## Supplementary Material



## Notes

Class-by-class derivation of the working equations for the EDPT2 method. This derivation covers the specific second-order contributions to both the neutral and ionic parts of the Heisenberg exchange interaction *J*. It is carried out for the two-electron-two-center case, with approximate corrections for higher-dimensional cases (e.g., the three-electron-three-center case) obtained by (i) accounting for missing contributions within the active space and (ii) including the missing two-electron interactions directly in the working equations (PDF)

## §

Institute of Physical and Theoretical Chemistry, Goethe University Frankfurt, Max-von-Laue-Straße 7, 60438 Frankfurt, Germany
