# Supplementary material for: Computation of Exchange Couplings by Means of an Exchange-Dedicated Perturbation Theory
Source: J Chem Theory Comput. 2025 Sep 4;21(18):8982–93. doi: 10.1021/acs.jctc.5c00733 (PMC12461945; doi:10.1021/acs.jctc.5c00733)
Supplement: Supplementary file 1 [file ct5c00733_si_001.pdf]

Supporting Information

Computation of exchange couplings by means of an exchange-dedicated perturbation theory

Michael Franz,[1] Frank Neese,[2*] Sabine Richert [1†*]

[1] *Institute of Physical Chemistry, University of Freiburg, Albertstraße 21, 79104 Freiburg, Germany*

[2] *Max-Planck-Institut für Kohlenforschung, Kaiser-Wilhelm-Platz 1, 45470 Mülheim an der Ruhr, Germany*

[†] *Present address: Institute of Physical and Theoretical Chemistry, Goethe University Frankfurt, Max-von-Laue-Straße 7, 60438 Frankfurt, Germany*

[*] E-mail: neese@kofo.mpg.de, sabine.richert@physchem.uni-freiburg.de

1 Derivation of the working equations

This section deals with the derivation of the specific working equations of the EDPT2 method. It is recommended to read the manuscript instead of the SI if the reader is interested in the derivation of the more general equations.
First, we will clarify some of the indices and entities:

- $t, u, v... \rightarrow$ active electron indices
- $i, j... \rightarrow$ internal orbital indices
- $a, b... \rightarrow$ virtual orbital indices
- $(pq|rs) \rightarrow$ two-electron integral in the chemist's notation
- $f^c_{pq} = h_{pq} + \sum_i 2(pq|ii) - (pi|iq) \rightarrow$ core-part of the CASSCF Fock matrix element

The derivation of the EDPT2 working equations starts by introducing the second-order correction to the exchange interaction J_{tu} between the orbitals t and u:

$$J^{(2)}_{tu,\text{neutral}} = (2c_\text{N}+1)\sum_{r\mu}\frac{\left\langle\phi_t\bar{\phi}_u\right|\hat{H}\left|\psi^r_\mu\right\rangle\left\langle\psi^r_\mu\right|\hat{H}\left|\phi_u\bar{\phi}_t\right\rangle}{\left\langle\phi_u\bar{\phi}_t\right|\hat{H}^{(0)}\left|\phi_t\bar{\phi}_u\right\rangle-\left\langle\phi^r_\mu\right|\hat{H}^{(0)}\left|\phi^r_\mu\right\rangle}, \tag{1}$$

$$\begin{aligned}J^{(2)}_{tu,\text{ionic}} =& 2c_\text{N}\sum_{\mu r}\frac{\left(\left\langle\phi_t\bar{\phi}_u\right|\hat{H}\left|\psi^r_\mu\right\rangle+\left\langle\phi_u\bar{\phi}_t\right|\hat{H}\left|\psi^r_\mu\right\rangle\right)\left(c_3\left\langle\phi_u\bar{\phi}_u\right|\hat{H}\left|\psi^r_\mu\right\rangle+c_4\left\langle\phi_t\bar{\phi}_t\right|\hat{H}\left|\psi^r_\mu\right\rangle\right)}{\left\langle\phi_u\bar{\phi}_t\right|\hat{H}^{(0)}\left|\phi_t\bar{\phi}_u\right\rangle-\left\langle\phi^r_\mu\right|\hat{H}^{(0)}\left|\phi^r_\mu\right\rangle}\\
&+\sum_{\mu r}\frac{2c_3c_4\left\langle\phi_u\bar{\phi}_u\right|\hat{H}\left|\psi^r_\mu\right\rangle\left\langle\phi_t\bar{\phi}_t\right|\hat{H}\left|\psi^r_\mu\right\rangle}{\left\langle\phi_u\bar{\phi}_t\right|\hat{H}^{(0)}\left|\phi_t\bar{\phi}_u\right\rangle-\left\langle\phi^r_\mu\right|\hat{H}^{(0)}\left|\phi^r_\mu\right\rangle}+\sum_{\mu r}\frac{c_3^2\left\langle\phi_u\bar{\phi}_u\right|\hat{H}\left|\psi^r_\mu\right\rangle^2}{\left\langle\phi_u\bar{\phi}_t\right|\hat{H}^{(0)}\left|\phi_t\bar{\phi}_u\right\rangle-\left\langle\phi^r_\mu\right|\hat{H}^{(0)}\left|\phi^r_\mu\right\rangle}\\
&+\sum_{\mu r}\frac{c_4^2\left\langle\phi_t\bar{\phi}_t\right|\hat{H}\left|\psi^r_\mu\right\rangle^2}{\left\langle\phi_u\bar{\phi}_t\right|\hat{H}^{(0)}\left|\phi_t\bar{\phi}_u\right\rangle-\left\langle\phi^r_\mu\right|\hat{H}^{(0)}\left|\phi^r_\mu\right\rangle}\\
&-\sum_{\mu r}\frac{\frac{1}{2}(c_3^2+c_4^2)\left(\left\langle\phi_t\bar{\phi}_u\right|\hat{H}\left|\psi^r_\mu\right\rangle^2+\left\langle\phi_u\bar{\phi}_t\right|\hat{H}\left|\psi^r_\mu\right\rangle^2\right)}{\left\langle\phi_u\bar{\phi}_t\right|\hat{H}^{(0)}\left|\phi_t\bar{\phi}_u\right\rangle-\left\langle\phi^r_\mu\right|\hat{H}^{(0)}\left|\phi^r_\mu\right\rangle},\end{aligned} \tag{2}$$

with:

$$J^{(2)}_{tu} = J^{(2)}_{tu,\text{neutral}} + J^{(2)}_{tu,\text{ionic}}. \tag{3}$$

The reference space is spanned by the determinants $\left|\phi_t\bar{\phi}_u\right\rangle$, $\left|\phi_u\bar{\phi}_t\right\rangle$, $\left|\phi_t\bar{\phi}_t\right\rangle$ and $\left|\phi_u\bar{\phi}_u\right\rangle$. The coefficients c_N, c_3 and c_4 correspond to the coefficients of the reference determinants in the predominantly singlet wavefunction, i.e $\psi^\text{S} = c_N(\left|\phi_t\bar{\phi}_u\right\rangle+\left|\phi_u\bar{\phi}_t\right\rangle)+c_3\left|\phi_u\bar{\phi}_u\right\rangle+c_4\left|\phi_t\bar{\phi}_t\right\rangle$. ψ^r_μ denote the excited Slater determinants, where r is a perturber class and μ addresses the specific Slater determinant of that perturber class. The zeroth-order

Hamiltonian is defined to be Dyall's Hamiltonian, which acts as the full Hamiltonian inside the active space (bielectronic interactions) and as the CASSCF Fock operator outside the active space.
In order to derive the specific working equations, we will insert the specific Slater determinants class by class into Equation (1) and Equation (2). The working equations are then derived using the Slater-Condon rules.

1.1 1h perturber class

We can define the following types of excited Slater determinants:

- $\left|\psi_\mu^r\right\rangle$: $\left|\phi_u\bar{\phi}_i\phi_t\bar{\phi}_u\right\rangle$, $\left|\phi_i\bar{\phi}_t\phi_t\bar{\phi}_u\right\rangle$, $\left|\phi_i\bar{\phi}_t\phi_u\bar{\phi}_u\right\rangle$, $\left|\phi_u\bar{\phi}_i\phi_t\bar{\phi}_t\right\rangle$

Inserting these Slater determinants into Equation (1) and Equation (2), we obtain:

$$\begin{aligned} J_{tu,\text{neutral}}^{(2),1h} = &-(4c_N+2)\sum_i \frac{[f_{iu}^c+(iu|tt)+(iu|uu)-(it|tu)](it|tu)}{\epsilon_i - f_{uu}^c-(tt|uu)-(uu|uu)+(tu|tu)} \\ &-(4c_N+2)\sum_i \frac{[f_{it}^c+(it|tt)+(it|uu)-(iu|ut)](iu|ut)}{\epsilon_i - f_{tt}^c-(tt|uu)-(tt|tt)+(tu|tu)} \end{aligned} \tag{4}$$

$$\begin{aligned} J_{tu,\text{ionic}}^{(2),1h} = &\sum_i \frac{4c_N[f_{iu}^c+(iu|tt)+(iu|uu)-2(it|tu)](-c_3[f_{it}^c+2(it|uu)-(iu|ut)]+c_4(iu|tu))}{\epsilon_i - f_{uu}^c-(tt|uu)-(uu|uu)+(tu|tu)} \\ &+\frac{2c_3^2[f_{it}^c+2(it|uu)-(iu|ut)]^2+2c_4^2(iu|ut)^2-4c_3c_4[f_{it}^c+2(it|uu)-(iu|ut)](iu|ut)}{\epsilon_i - f_{uu}^c-(tt|uu)-(uu|uu)+(tu|tu)} \\ &-\frac{(c_3^2+c_4^2)([f_{iu}^c+(iu|tt)+(iu|uu)-(it|tu)]^2+(it|tu)^2)}{\epsilon_i - f_{uu}^c-(tt|uu)-(uu|uu)+(tu|tu)} \\ &+\sum_i \frac{4c_N[f_{it}^c+(it|tt)+(it|uu)-2(iu|ut)](-c_3(it|tu)+c_4[f_{iu}^c+2(iu|tt)-(it|tu)])}{\epsilon_i - f_{tt}^c-(tt|uu)-(tt|tt)+(tu|tu)} \\ &+\frac{2c_3^2(it|tu)^2+2c_4^2[f_{iu}^c+2(iu|tt)-(it|tu)]^2-4c_3c_4[f_{iu}^c+2(iu|tt)-(it|tu)](it|tu)}{\epsilon_i - f_{tt}^c-(tt|uu)-(tt|tt)+(tu|tu)} \\ &-\frac{(c_3^2+c_4^2)([f_{it}^c+(it|tt)+(it|uu)-(iu|ut)]^2+(iu|ut)^2)}{\epsilon_i - f_{uu}^c-(tt|uu)-(uu|uu)+(tu|tu)} \end{aligned} \tag{5}$$

1.2 1p perturber class

We can define the following types of excited Slater determinants:

- $\left|\psi_\mu^r\right\rangle$: $\left|\phi_t\bar{\phi}_a\right\rangle$, $\left|\phi_a\bar{\phi}_u\right\rangle$, $\left|\phi_u\bar{\phi}_a\right\rangle$, $\left|\phi_a\bar{\phi}_t\right\rangle$

Inserting these Slater determinants into Equation (1) and Equation (2), we obtain:

$$J^{(2),1p}_{tu,\text{neutral}} = (4c_N+2)\sum_a \frac{[f^c_{ua}+(ua|tt)](ut|ta)}{f^c_{uu}-\epsilon_a+(tt|uu)} + \frac{[f^c_{ta}+(ta|uu)](tu|ua)}{f^c_{tt}-\epsilon_a+(tt|uu)} \tag{6}$$

$$\begin{aligned} J^{(2),1p}_{tu,\text{ionic}} =& \sum_a \frac{4c_N[f^c_{ua}+(ua|tt)+(ut|ta)](c_3(ut|ta)+c_4[f^c_{ta}+(ta|tt)])}{f^c_{uu}-\epsilon_a+(tt|uu)} \\ &+ \frac{2c_3^2(ut|ta)^2+2c_4^2[f^c_{ta}+(ta|tt)]^2+4c_3c_4(ut|ta)[f^c_{ta}+(ta|tt)]}{f^c_{uu}-\epsilon_a+(tt|uu)} \\ &- \frac{(c_3^2+c_4^2)([f^c_{ua}+(ua|tt)]^2+(ut|ta)^2)}{f^c_{uu}-\epsilon_a+(tt|uu)} \\ &+ \sum_a \frac{4c_N[f^c_{ta}+(ta|uu)+(tu|ua)](c_3[f^c_{ua}+(ua|uu)]+c_4(ut|ta))}{f^c_{tt}-\epsilon_a+(tt|uu)} \\ &+ \frac{2c_3^2[f^c_{ua}+(ua|uu)]^2+2c_4^2(ut|ta)^2+4c_3c_4[f^c_{ua}+(ua|uu)](ut|ta)}{f^c_{tt}-\epsilon_a+(tt|uu)} \\ &- \frac{(c_3^2+c_4^2)([f^c_{ta}+(ta|uu)]^2+(tu|ua)^2)}{f^c_{tt}-\epsilon_a+(tt|uu)} \end{aligned} \tag{7}$$

1.3 1h-1p perturber class: internal → virtual single excitations

We can define the following types of excited Slater determinants:

- $\left|\psi^r_\mu\right\rangle$: $\left|\phi_i\bar{\phi}_a\phi_t\bar{\phi}_u\right\rangle$, $\left|\phi_a\bar{\phi}_i\phi_t\bar{\phi}_u\right\rangle$, $\left|\phi_i\bar{\phi}_a\phi_u\bar{\phi}_t\right\rangle$, $\left|\phi_a\bar{\phi}_i\phi_u\bar{\phi}_t\right\rangle$

Inserting these Slater determinants into Equation (1) and Equation (2), we obtain:

$$\begin{aligned} J^{(2),\text{SE}}_{tu,\text{ionic}} =& \sum_{ia} \frac{4c_n[f^c_{ia}+(ia|tt)+(ia|uu)-(iu|ua)](c_3(tu|ia)+c_4[(tu|ia)-(ta|ui)]}{\epsilon_i-\epsilon_a} \\ &+ \frac{4c_3c_4(tu|ia)[(tu|ia)-(ta|ui)]+2c_3^2(tu|ia)^2+2c_4^2[(tu|ia)-(ta|ui)]^2}{\epsilon_i-\epsilon_a} \\ &- \frac{(c_3^2+c_4^2)[f^c_{ia}+(ia|tt)+(ia|uu)-(iu|ua)]^2}{\epsilon_i-\epsilon_a} \\ &+ \sum_{ia} \frac{4c_n[f^c_{ia}+(ia|tt)+(ia|uu)-(it|ta)](c_4(tu|ia)+c_3[(tu|ia)-(ti|ua)]}{\epsilon_i-\epsilon_a} \\ &+ \frac{4c_3c_4(tu|ia)[(tu|ia)-(ti|ua)]+2c_4^2(tu|ia)^2+2c_3^2[(tu|ia)-(ti|ua)]^2}{\epsilon_i-\epsilon_a} \\ &- \frac{(c_3^2+c_4^2)[f^c_{ia}+(ia|tt)+(ia|uu)-(it|ta)]^2}{\epsilon_i-\epsilon_a} \end{aligned} \tag{8}$$

Note, that there is only an ionic contribution of this perturber class to the exchange interaction.

1.4 1h-1p perturber class: dynamic spin polarisation

We can define the following types of excited Slater determinants:

- $\left|\psi_\mu^r\right\rangle$: $\left|\phi_i\bar{\phi}_t\phi_a\bar{\phi}_u\right\rangle$, $\left|\phi_u\bar{\phi}_i\phi_t\bar{\phi}_a\right\rangle$

Inserting these Slater determinants into Equation (1) and Equation (2), we obtain:

$$J_{tu,\text{neutral}}^{(2),\text{DSP}} = -(4c_N^2+2)\sum_{ia}\frac{(it|ta)(iu|ua)}{\epsilon_i-\epsilon_a+(tu|tu)} \tag{9}$$

$$\begin{aligned} J_{tu,\text{ionic}}^{(2),\text{DSP}} =& \sum_{ia}\frac{4c_N[(it|ta)-(iu|ua)][c_3(ti|ua)-c_4(ta|ui)]+4c_3c_4(ti|ua)(ta|ui)}{\epsilon_i-\epsilon_a+(tu|tu)} \\ &+\frac{2c_3^2(ti|ua)^2+2c_4^2(ta|ui)^2-(c_3^2+c_4^2)[(it|ta)^2+(iu|ua)^2]}{\epsilon_i-\epsilon_a+(tu|tu)} \end{aligned} \tag{10}$$

1.5 1h-1p perturber class: dynamic charge polarisation

We can define the following types of excited Slater determinants:

- $\left|\psi_\mu^r\right\rangle$: $\left|\phi_i\bar{\phi}_a\phi_u\bar{\phi}_u\right\rangle$, $\left|\phi_a\bar{\phi}_i\phi_u\bar{\phi}_u\right\rangle$, $\left|\phi_i\bar{\phi}_a\phi_t\bar{\phi}_t\right\rangle$, $\left|\phi_a\bar{\phi}_i\phi_t\bar{\phi}_t\right\rangle$

Inserting these Slater determinants into Equation (1) and Equation (2), we obtain:

$$\begin{aligned} J_{tu,\text{neutral}}^{(2),\text{DCP}} = (4c_N^2+2)\sum_{ia} & \frac{(tu|ia)[(tu|ia)-(ui|ta)]}{\epsilon_i-\epsilon_a+f_{tt}^c-f_{uu}^c+(tu|tu)-(uu|uu)} \\ &+\frac{(tu|ia)[(tu|ia)-(ti|ua)]}{\epsilon_i-\epsilon_a+f_{uu}^c-f_{tt}^c+(tu|tu)-(tt|tt)} \end{aligned} \tag{11}$$

$$\begin{aligned} J_{tu,\text{ionic}}^{(2),\text{DCP}} =& \sum_{ia}\frac{4c_Nc_3[2(tu|ia)-(iu|ta)][f_{ia}^c+2(ia|uu)-(iu|ua)]}{\epsilon_i-\epsilon_a+f_{tt}^c-f_{uu}^c+(tu|tu)-(uu|uu)} \\ &+\frac{2c_3^2[f_{ia}^c+2(ia|uu)-(iu|ua)]^2-(c_3^2+c_4^2)[(tu|ia)^2+[(tu|ia)-(iu|ta)]^2]}{\epsilon_i-\epsilon_a+f_{tt}^c-f_{uu}^c+(tu|tu)-(uu|uu)} \\ &+\frac{4c_Nc_4[2(tu|ia)-(it|ua)][f_{ia}^c+2(ia|tt)-(it|ta)]}{\epsilon_i-\epsilon_a+f_{uu}^c-f_{tt}^c+(tu|tu)-(tt|tt)} \\ &+\frac{2c_4^2[f_{ia}^c+2(ia|tt)-(it|ta)]^2-(c_3^2+c_4^2)[(tu|ia)^2+[(tu|ia)-(it|ua)]^2]}{\epsilon_i-\epsilon_a+f_{uu}^c-f_{tt}^c+(tu|tu)-(tt|tt)} \end{aligned} \tag{12}$$

1.6 2h perturber class

We can define the following type of excited Slater determinants:

- $\left|\psi_\mu^r\right\rangle$: $\left|\phi_u\bar{\phi}_t\phi_t\bar{\phi}_u\right\rangle$,

Inserting these Slater determinants into Equation (1) and Equation (2), we obtain:

$$J_{tu,\text{neutral}}^{(2),2h} = (2c_N^2+1)\sum_{ij}\frac{(ti|uj)(tj|ui)}{\epsilon_i+\epsilon_j-f_{uu}^c-f_{tt}^c-3(tt|uu)+2(tu|tu)-(tt|tt)-(uu|uu)} \tag{13}$$

$$\begin{aligned} J_{tu,\text{ionic}}^{(2),2h} =& \sum_{ij}\frac{-2c_N[(tj|ui)+(ti|uj)][c_3(it|tj)+c_4(iu|uj)]+2c_3c_4(it|tj)(iu|uj)}{\epsilon_i+\epsilon_j-f_{uu}^c-f_{tt}^c-3(tt|uu)+2(tu|tu)-(tt|tt)-(uu|uu)} \\ &+\frac{c_3^2(it|tj)^2+c_4^2(iu|uj)^2-\frac{1}{2}(c_3^2+c_4^2)[(tj|ui)^2+(ti|uj)^2]}{\epsilon_i+\epsilon_j-f_{uu}^c-f_{tt}^c-3(tt|uu)+2(tu|tu)-(tt|tt)-(uu|uu)} \end{aligned} \tag{14}$$

1.7 2p perturber class

We can define the following type of excited Slater determinants:

- $\left|\psi_\mu^r\right\rangle$: $\left|\phi_a\bar{\phi}_b\right\rangle$,

Inserting these Slater determinants into Equation (1) and Equation (2), we obtain:

$$J_{tu,\text{neutral}}^{(2),2p} = (2c_N^2+1)\sum_{ab}\frac{(ta|ub)(tb|ua)}{f_{uu}^c+f_{tt}^c-\epsilon_a-\epsilon_b+(tu|tu)} \tag{15}$$

$$\begin{aligned} J_{tu,\text{ionic}}^{(2),2p} =& \sum_{ab}\frac{2c_N[(ta|ub)+(tb|ua)][c_3(au|ub)+c_4(at|tb)]+2c_3c_4(au|ub)(at|tb)}{f_{uu}^c+f_{tt}^c-\epsilon_a-\epsilon_b+(tu|tu)} \\ &+\frac{c_3^2(au|ub)^2+c_4^2(at|tb)^2-\frac{1}{2}(c_3^2+c_4^2)[(ta|ub)^2+(tb|ua)^2]}{f_{uu}^c+f_{tt}^c-\epsilon_a-\epsilon_b+(tu|tu)} \end{aligned} \tag{16}$$

1.8 2h-1p perturber class

We can define the following types of excited Slater determinants:

- $\left|\psi_\mu^r\right\rangle$: $\left|\phi_a\bar{\phi}_i\phi_u\bar{\phi}_j\phi_t\bar{\phi}_u\right\rangle$, $\left|\phi_i\bar{\phi}_a\phi_u\bar{\phi}_j\phi_t\bar{\phi}_u\right\rangle$, $\left|\phi_i\bar{\phi}_a\phi_j\bar{\phi}_t\phi_t\bar{\phi}_u\right\rangle$, $\left|\phi_a\bar{\phi}_i\phi_j\bar{\phi}_t\phi_t\bar{\phi}_u\right\rangle$, $\left|\phi_i\bar{\phi}_a\phi_j\bar{\phi}_u\phi_u\bar{\phi}_t\right\rangle$, $\left|\phi_a\bar{\phi}_i\phi_j\bar{\phi}_u\phi_u\bar{\phi}_t\right\rangle$, $\left|\phi_a\bar{\phi}_i\phi_t\bar{\phi}_j\phi_u\bar{\phi}_t\right\rangle$, $\left|\phi_i\bar{\phi}_a\phi_t\bar{\phi}_j\phi_u\bar{\phi}_t\right\rangle$

Inserting these Slater determinants into Equation (1) and Equation (2), we obtain:

$$
\begin{aligned}
J^{(2),2h-1p}_{tu,\text{ionic}} =& 2\sum_{ija} \frac{2c_N[(ia|ju)-(ja|iu)][-c_3[(ia|jt)-(ja|it)]+c_3^2[(ia|jt)-(ja|it)]^2}{\epsilon_i+\epsilon_j-f^c_{uu}-\epsilon_a-(tt|uu)-(uu|uu)+(tu|tu)} \\
&- \frac{\frac{1}{2}(c_3^2+c_4^2)[(ia|ju)-(ja|iu)]^2}{\epsilon_i+\epsilon_j-f^c_{uu}-\epsilon_a-(tt|uu)-(uu|uu)+(tu|tu)} \\
&+ \frac{-2c_Nc_3(ia|ju)(ia|jt)+c_3^2(ia|jt)^2-\frac{1}{2}(c_3^2+c_4^2)(ia|ju)^2}{\epsilon_i+\epsilon_j-f^c_{uu}-\epsilon_a-(tt|uu)-(uu|uu)+(tu|tu)} \\
&+ \frac{2c_N[(ia|jt)-(ja|it)][-c_4[(ia|ju)-(ja|iu)]+c_4^2[(ia|ju)-(ja|iu)]^2}{\epsilon_i+\epsilon_j-f^c_{tt}-\epsilon_a-(tt|uu)-(tt|tt)+(tu|tu)} \\
&- \frac{\frac{1}{2}(c_3^2+c_4^2)[(ia|jt)-(ja|it)]^2}{\epsilon_i+\epsilon_j-f^c_{tt}-\epsilon_a-(tt|uu)-(tt|tt)+(tu|tu)} \\
&+ \frac{-2c_Nc_4(ia|jt)(ia|ju)+c_3^2(ia|ju)^2-\frac{1}{2}(c_3^2+c_4^2)(ia|jt)^2}{\epsilon_i+\epsilon_j-f^c_{tt}-\epsilon_a-(tt|uu)-(tt|tt)+(tu|tu)}
\end{aligned}
\tag{17}
$$

Note, that there is only an ionic contribution of this perturber class to the exchange interaction.

1.9 1h-2p perturber class

We can define the following types of excited Slater determinants:

- $\left|\psi^r_\mu\right\rangle$: $\left|\phi_i\bar{\phi}_b\phi_a\bar{\phi}_u\right\rangle$, $\left|\phi_b\bar{\phi}_i\phi_a\bar{\phi}_u\right\rangle$, $\left|\phi_b\bar{\phi}_i\phi_t\bar{\phi}_a\right\rangle$, $\left|\phi_i\bar{\phi}_b\phi_t\bar{\phi}_a\right\rangle$, $\left|\phi_i\bar{\phi}_a\phi_u\bar{\phi}_b\right\rangle$, $\left|\phi_a\bar{\phi}_i\phi_b\bar{\phi}_t\right\rangle$, $\left|\phi_a\bar{\phi}_i\phi_u\bar{\phi}_b\right\rangle$, $\left|\phi_i\bar{\phi}_b\phi_a\bar{\phi}_t\right\rangle$

Inserting these Slater determinants into Equation (1) and Equation (2), we obtain:

$$
\begin{aligned}
J^{(2),2h-1p}_{tu,\text{ionic}} =& 2\sum_{ija} \frac{2c_N[(ib|ta)-(ia|tb)][c_3[(ib|ua)-(ia|ub)]+c_3^2[(ib|ua)-(ia|ub)]^2}{\epsilon_i+f^c_{tt}-\epsilon_a-\epsilon_b+(tt|uu)} \\
&- \frac{\frac{1}{2}(c_3^2+c_4^2)[(ib|ta)-(ia|tb)]^2}{\epsilon_i+f^c_{tt}-\epsilon_a-\epsilon_b+(tt|uu)} \\
&+ \frac{2c_Nc_3(ib|ta)(ib|ua)+c_3^2(ib|ua)^2-\frac{1}{2}(c_3^2+c_4^2)(ib|ta)^2}{\epsilon_i+f^c_{tt}-\epsilon_a-\epsilon_b+(tt|uu)} \\
&+ \frac{2c_N[(ib|ua)-(ia|ub)][c_4[(ib|ta)-(ia|tb)]+c_4^2[(ib|ta)-(ia|tb)]^2}{\epsilon_i+f^c_{uu}-\epsilon_a-\epsilon_b+(tt|uu)} \\
&- \frac{\frac{1}{2}(c_3^2+c_4^2)[(ib|ua)-(ia|ub)]^2}{\epsilon_i+f^c_{uu}-\epsilon_a-\epsilon_b+(tt|uu)} \\
&+ \frac{2c_Nc_4(ib|ua)(ib|ta)+c_3^2(ib|ta)^2-\frac{1}{2}(c_3^2+c_4^2)(ib|ua)^2}{\epsilon_i+f^c_{uu}-\epsilon_a-\epsilon_b+(tt|uu)}
\end{aligned}
\tag{18}
$$

Note, that there is only an ionic contribution of this perturber class to the exchange interaction.

1.10 Additional corrections within the active space

Since the working equations are derived for the two-electron-two-center case, there will be missing terms that arise solely from the active space in the case that there are more than two active electrons and orbitals. The missing terms are derived on the three-electron-three-center case. However, we only consider those terms that involve single excitations. Furthermore, we only consider the neutral contribution to the exchange interaction $J^{(2)}_{tu,\mathrm{neutral}}$. Note, that the contribution of the additional corrections are expected to be small since the excited determinant space is limited within the CAS.
We can define the following types of excited Slater determinants:

- $\left|\psi^r_\mu\right\rangle$: $\left|\phi_t\bar{\phi}_u\phi_u\right\rangle$, $\left|\phi_t\bar{\phi}_t\phi_u\right\rangle$, $\left|\phi_t\bar{\phi}_v\phi_v\right\rangle$, $\left|\phi_u\bar{\phi}_v\phi_v\right\rangle$

In this case, $J^{(2)}_{tu,\mathrm{neutral}}$ is computed as:

$$J^{(2)}_{tu,\mathrm{neutral}} = (2c_\mathrm{N}+1)\sum_{r\mu}\frac{\left\langle\phi_t\bar{\phi}_u\phi_v\right|\hat{H}\left|\psi^r_\mu\right\rangle\left\langle\psi^r_\mu\right|\hat{H}\left|\phi_u\bar{\phi}_t\phi_v\right\rangle}{\left\langle\phi_u\bar{\phi}_t\phi_v\right|\hat{H}^{(0)}\left|\phi_t\bar{\phi}_u\phi_v\right\rangle-\left\langle\phi^r_\mu\right|\hat{H}^{(0)}\left|\phi^r_\mu\right\rangle}, \tag{19}$$

Inserting the excited determinants into Equation (19), we obtain:

$$\begin{aligned} J^{(2),\mathrm{add}}_{tu,\mathrm{neutral}} = &\sum_v \frac{-[f^c_{uv}+(uv|tt)+(uv|uu)-(ut|tv)](ut|tv)}{f^c_{vv}-f^c_{uu}+(uu|vv)-(uu|uu)} \\ &+\frac{-[f^c_{tv}+(tv|tt)+(tv|uu)-(tu|uv)](tu|uv)}{f^c_{vv}-f^c_{tt}+(tt|vv)-(tt|tt)} \\ &+\frac{[f^c_{uv}+(uv|vv)+(uv|tt)](ut|tv)}{f^c_{uu}-f^c_{vv}+(uu|vv)-(vv|vv)} \\ &+\frac{[f^c_{tv}+(tv|vv)+(tv|uu)](tu|uv)}{f^c_{tt}-f^c_{vv}+(tt|vv)-(vv|vv)} \end{aligned} \tag{20}$$

1.11 Two-electron interactions from missing active electrons

Since the working equations were derived for the two-electron-two-center case, there will be missing Coulomb and exchange potentials due to the presence of additional electrons in the active space in the case that the active space contains more than two electrons and orbitals.
We may demonstrate the effect of an additional electron in the active space by deriving two different single-excitation Hamiltonian matrix elements:

$$\left\langle \phi_t \bar{\phi}_u \right| \hat{H} \left| \phi_u \bar{\phi}_u \right\rangle = f^c_{tu} + (tu|uu) \tag{21}$$

$$\left\langle \phi_t \bar{\phi}_u \phi_v \right| \hat{H} \left| \phi_u \bar{\phi}_u \phi_v \right\rangle = f^c_{tu} + (tu|uu) + (tu|vv) - (tv|vu) \tag{22}$$

The Hamiltonian matrix element with the additional active electron includes a Coulomb and an exchange potential arising from that additional electron. In order to account for this effect, all single excitation terms in the working equations are modified by inclusion of a summation of the Coulomb potentials over all missing active electrons assuming only one electron in one orbital. The exchange potentials are neglected. In that case a single excitation Hamiltonian matrix element is computed as:

$$\left\langle \phi_t \bar{\phi}_u \phi_v ... \phi_w \right| \hat{H} \left| \phi_u \bar{\phi}_u \phi_v ... \phi_w \right\rangle = f^c_{tu} + (tu|uu) + \sum_v^w (tu|vv), \tag{23}$$

where the dots indicite additional active electrons. Note, that additional electrons in the active space would also alternate the denominators in the working equations. However, no modification of the denominators is considered.
